# The Expanding Role of Aquaporin-1, Aquaporin-3 and Aquaporin-5 as Transceptors: Involvement in Cancer Development and Potential Druggability

**DOI:** 10.3390/ijms26031330

**Published:** 2025-02-04

**Authors:** Catarina Pimpão, Inês V. da Silva, Graça Soveral

**Affiliations:** 1 Research Institute for Medicines (iMed.ULisboa), Faculty of Pharmacy, Universidade de Lisboa, 1649-003 Lisboa, Portugal; 2 Department of Pharmaceutical Sciences and Medicines, Faculty of Pharmacy, Universidade de Lisboa, 1649-003 Lisboa, Portugal

**Keywords:** aquaporins, cancer, signaling pathways, transporters, modulators

## Abstract

Aquaporins (AQPs) are transmembrane proteins that facilitate the transport of water and small solutes, including glycerol, hydrogen peroxide and ions, across cell membranes. Beyond their established physiological roles in water regulation and metabolic processes, AQPs also exhibit receptor-like signaling activities in cancer-associated signaling pathways, integrating the dual roles of transporters and receptors, hence functioning as transceptors. This dual functionality underpins their critical involvement in cancer biology, where AQPs play key roles in promoting cell proliferation, migration, and invasion, contributing significantly to carcinogenesis. Among the AQPs, AQP1, AQP3 and AQP5 have been consistently identified as being aberrantly expressed in various tumor types. Their overexpression is strongly associated with tumor progression, metastasis, and poor patient prognosis. This review explores the pivotal roles of AQP1, AQP3 and AQP5 as transceptors in cancer biology, underscoring their importance as pharmacological targets. It highlights the urgent need for the development of effective modulators to target these AQPs, offering a promising avenue to enhance current therapeutic approaches for cancer treatment.

## 1. Introduction

Aquaporins (AQPs) are transmembrane protein channels that facilitate the bidirectional transport of water, glycerol and other small non-charged solutes across cell membranes, being involved in water and energy homeostasis [1]. These proteins can be found in all living organisms, including archaea, eubacteria, fungi, plants and animals [2]. In humans, 13 AQP isoforms (AQP0-12) identified are widely distributed in the body and differentially expressed in each tissue and cell type [3]. According to their primary structure and pore selectivity, AQPs are classified into three subgroups: (1) orthodox or classical aquaporins (AQP0, AQP1, AQP2, AQP4, AQP5, AQP6 and AQP8), that are considered primarily selective to water; (2) aquaglyceroporins (AQP3, AQP7, AQP9 and AQP10) that, besides water, can also transport glycerol and other small neutral solutes; and (3) subcellular or unorthodox aquaporins (AQP11 and AQP12) [4] with a distinct evolutionary pathway, localized in intracellular membranes and with their permeability still uncertain [5], although AQP11 was found to permeate both water and glycerol [6]. A few of the above-mentioned isoforms can also transport ammonia (ammoniaporins) [7] and gases such as CO_2_ [8], O_2_ [9] and NO [10]. An overlapping subgroup, the peroxiporins (AQP0, AQP1, AQP3, AQP5, AQP8, AQP9, AQP11), comprises isoforms that facilitate the diffusion of hydrogen peroxide (H_2_O_2_) across membranes [11,12].

**Figure 1:**
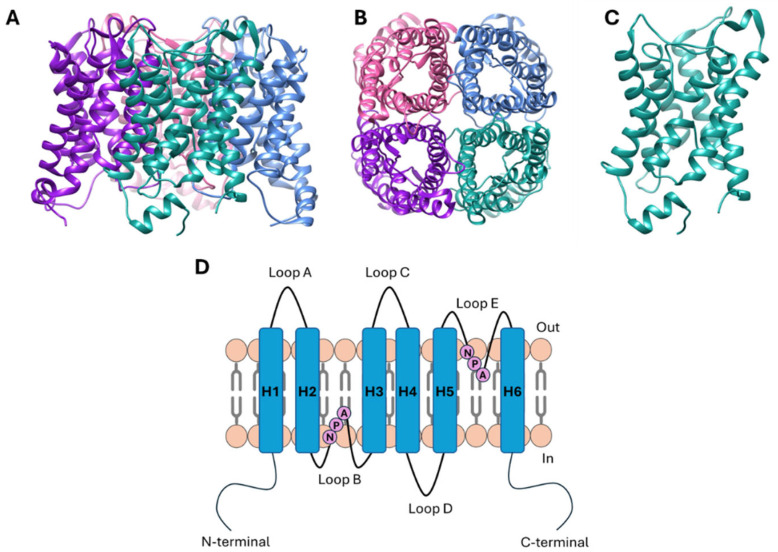
General three-dimensional structure of aquaporins. (**A**) Side and (**B**) top view of human AQP5 tetrameric structure, depicted in ribbon representation. (**C**) Side view of AQP5 monomer hourglass structure. Picture edited in UCSF Chimera software 1.15 (PDB code: 3D9S). (**D**) Schematic representation of aquaporin topology within membrane. Each monomer comprises six transmembrane helices (H1–H6) connected by five loops (A–E). Loops B and E fold into membrane, constituting two helices containing conserved NPA (Asn-Pro-Ala) motifs, with both N-terminal and C-terminal domains located in cytoplasm.

AQPs are assembled as homotetramers in cell membranes, where each monomer acts as an independent pore (Figure 1A,B) [13]. Each AQP monomer has approximately 28 kDa and is formed by six transmembrane helices and five connecting loops, with loop B and E containing the conserved Asn-Pro-Ala (NPA) motifs that form short α-helices that fold into the membrane to form the pore, leading to an hourglass structure (Figure 1C,D) [13,14]. AQP monomers have a cytoplasmic N- and C-terminal and present two main constriction sites responsible for pore selectivity and proton exclusion. The first one corresponds to the aromatic/arginine (ar/R) selectivity filter (SF), near the extracellular pore entrance, which determines the size of molecules allowed to be permeated, being responsible for the selectivity to water and/or small neutral solutes and forming the narrowest region of the pore (2.8 Å diameter pore for orthodox AQPs and >3.4 Å for aquaglyceroporins). The second constriction site is located at the center of the channel and consists of two half-helices containing the conserved NPA motifs that generate a dipole moment, creating a positive electrostatic barrier that prevents the passage of positively charged ions through the pore [15,16,17,18].

## 2. Physiological Roles of AQPs and Their Implication in Cancer

The investigation of aquaporins as therapeutic targets is emerging as a hot topic within the scientific community. Studies on AQP-knockout (KO) mice have provided information about the importance of AQPs in physiology such as renal water reabsorption, brain water homeostasis and metabolism but also in their implication in a broad range of disorders such as metabolic disorders, inflammation and cancer [19,20,21,22].

Numerous studies have reported that AQPs play key roles in tumor biology, facilitating cancer cell migration, proliferation and angiogenesis [23]. In particular, AQP1, AQP3 and AQP5, in addition to their physiological role in transepithelial water transport, skin hydration and fluid secretion [1,19,24,25], were found to be overexpressed in several types of cancer [26,27,28,29], such as colon cancer [30], hepatocellular carcinoma [31] and pancreatic ductal adenocarcinoma [32], being correlated with tumor progression, metastasis and poor patient prognosis, and implying their importance as prognostic markers and therapeutic targets for anticancer drug discovery. AQP4 has been consistently linked to glioblastoma progression contributing not only to cancer migration and invasion [33,34] but also influencing the glioma tumor microenvironment [35]. Similarly, AQP9 regulates the tumor microenvironment in kidney cancer [36], hepatocellular carcinoma [37] and colon cancer, where it modulates the polarization of tumor-associated macrophages [38]. Although less studied, AQP2, AQP6, AQP7 and AQP8 are also implicated in tumorigenesis. AQP2 is overexpressed in endometrial carcinoma by estrogen, promoting cancer migration, invasion and adhesion [39]. AQP6 and AQP7 can increase the resistance to oxidative stress in mesothelioma cells [40] and breast cancer cells, respectively, with AQP7 also altering cancer cell metabolism [41]. AQP8 overexpression reduces colorectal cancer growth and spread [42] but enhances cancer cell proliferation, migration and invasion in gliomas and cervical cancer [43,44].

Considering the number of studies reporting the consistent implication of AQP1, AQP3 and AQP5 in the development of multiple cancer types and their strong association with signaling pathways, this review is focused on the critical roles of these AQPs as transceptors involved in cancer progression.

### 2.1. AQP1, AQP3 and AQP5: Key Players in Cancer Progression

AQP1-null mice implanted with melanoma cells showed impaired tumor growth, reduced angiogenesis and decreased cell migration. These findings underline the crucial role of endothelial AQP1 in facilitating cancer cell migration [45].

**Figure 2:**
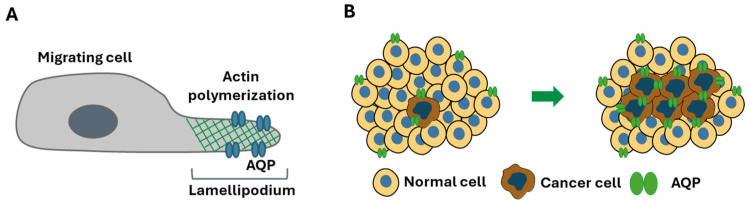
Role of aquaporins (AQPs) in cancer cell migration and proliferation. (**A**) AQPs are involved in cancer cell migration by facilitating fast water fluxes, generating local hydrostatic pressure that allows lamellipodium expansion for actin polymerization. (**B**) AQPs are overexpressed in cancer cells, influencing their proliferation through regulation of glycerol metabolism and modulation of signaling pathways such as PI3K, ERK and Wnt.

A mechanism for the involvement of AQPs in cell migration has been proposed based on their role as membrane channels and their polarization at the leading edge of the lamellipodium in migrating cells. Local osmotic gradients created by the cleavage of actin and ion uptake at the lamellipodium tip induce fast water fluxes mediated by AQPs, required for the rapid changes in cell shape. These events cause an increase in the local hydrostatic pressure, leading to the expansion of the lamellipodium and creating spaces for actin polymerization and consequent cell migration (Figure 2) [46].

Cancer cell migration can contribute to tumor cell infiltration in the surrounding tissue and consequently lead to metastasis [23]. Since AQP1 is overexpressed in tumor endothelial cells, this protein is crucial for angiogenesis, endothelial cell migration and tumor growth, enhancing tumor infiltration and spread. This correlation was verified in studies with AQP1-null mice with melanoma [45,47] and breast cancer [48,49] as well as in colon and lung cancer cell lines [26,50]. A possible interaction between AQP1 and the vascular endothelial growth factor (VEGF) signaling pathway was reported, implicating AQP1 in tumor angiogenesis and endometrial adenocarcinoma progression [51,52]. In addition, AQP1 was shown to contribute to hypoxia-inducible angiogenesis in retinal vascular endothelial cells through a VEGF-independent mechanism [53]. Interestingly, hypoxia-induced AQP1 upregulation was correlated with the p38 mitogen activated protein kinase (MAPK) signaling pathway in prostate cancer cells [54].

AQP1 has also been related to epithelial–mesenchymal transition (EMT), a cellular process during which epithelial cells undergo phenotypic changes, losing their cell–cell adhesion and cell polarity while acquiring mesenchymal features that include invasiveness and cell motility [52]. AQP1 overexpression in lung adenocarcinoma was associated with EMT markers: loss of E-cadherin (epithelial marker) and increased expression of vimentin (mesenchymal marker), indicating the involvement of AQP1 in EMT and consequent invasive potential [55].

AQP3 is the aquaglyceroporin most associated with tumor progression, being overexpressed in different types of cancer such as skin [56,57], lung [58], colon [59], pancreatic [32] and liver cancer [60]. AQP3-null mice were found to be resistant to the development of skin tumors after exposure to a skin tumor initiator and phorbol ester promoter, exhibiting a reduction in epidermal cell proliferation. In addition, a notable decrease in glycerol and its metabolite glycerol-3-phosphate was detected, correlating with a reduction in ATP levels. After oral glycerol administration, ATP levels and epidermal cell proliferation were reestablished, suggesting that AQP3-facilitated glycerol permeability contributes to the generation of ATP and is implicated in tumor growth [1,56]. In several tumors, AQP3 expression was correlated with the activation of signaling pathways that promote cancer cell proliferation, migration and invasion [61]. For instance, human epidermal growth factor (EGF) upregulates AQP3 expression, thereby enhancing colorectal cancer cell migration via the phosphoinositide 3-kinase (PI3K)/Akt signaling pathway, being also associated with metastasis in colorectal cancer patients [62]. Likewise, AQP3 was reported to promote EMT through the activation of the PI3K/Akt/SNAIL signaling pathway in gastric cancer cells [63] and was found to contribute to stem-like properties, facilitating tumor growth via the Wnt/glycogen synthase kinase-3 beta (GSK-3β)/β-catenin signaling pathway [64]. In pancreatic cancer cells, AQP3 was found to promote tumor proliferation through the activation of the mammalian target of rapamycin (mTOR) signaling pathway [28]. Additionally, AQP3 overexpression activates the extracellular signal-regulated kinase (ERK)1/2 pathway in prostate cancer cells, leading to increased expression of matrix metalloproteinase-3 (MMP-3), which stimulates cell motility and invasion, further reinforcing AQP3’s contribution to cancer aggressiveness [65]. Furthermore, AQP3 suppression in esophageal and oral squamous cell carcinoma cells led to reduced cell adhesion and increased cell death through the inhibition of focal adhesion kinase (FAK) phosphorylation, decreasing the phosphorylation of ERK and MAPK pathways [66]. These findings highlight AQP3 as a critical mediator of EMT, tumor growth and metastasis, through its role in glycerol permeation, ATP production and the activation of key signaling pathways that contribute to tumor progression across various cancer types.

AQP5 involvement in tumor initiation and progression has been strongly linked with the activation of signaling pathways that can contribute to cancer cell migration, invasion and proliferation [67]. For example, AQP5 overexpression in colon cancer cells promotes cell proliferation via the Ras signaling pathway, probably mediated by the phosphorylation of the PKA consensus site of AQP5, and contributes to EMT by increasing phosphorylated Smad2/3 levels [68,69,70]. AQP5 silencing decreases cell invasion by modulating EMT-related proteins in colon cancer and hepatocellular carcinoma (HCC), but also through the downregulation of Wnt/β-catenin and the nuclear factor-kappa B (NF-ĸB) signaling pathways, respectively [71,72]. Additionally, AQP5 stimulates lung cancer cell proliferation and migration through the modulation of the epidermal growth factor receptor (EGFR)/ERK/p38 MAPK signaling pathway, while also upregulating the proliferation marker c-myc and increasing MUC5AC mucin production, revealing that AQP5 contributes to a higher metastatic and invasive potential [29]. In addition, the incubation of rat astrocytes in hypotonic media increased AQP5 expression, potentially regulated by the p38 MAPK pathway [73]. Thus, these findings indicate that AQP5 overexpression in cancer cells boosts their metastatic and invasive potential by modulating key signal transduction pathways, contributing to tumor progression.

In colorectal cancer progression, AQP1 and AQP5 expression was found to be induced in the early stage of the disease and maintained stability through the late stage of cancer development [74]. Interestingly, AQP3 and AQP5 expression patterns vary during the progression of pancreatic ductal adenocarcinoma. AQP3 expression levels are increased with cancer development from the early to late stages of the tumor, while AQP5 expression is augmented in the early stage but almost undetectable in later stages, showing its potential as a biomarker of this disease. In the same study, AQP3 and AQP5 were associated with increased levels of EGFR, proliferation marker Ki-67, cytokeratin 7 and vimentin, while E-cadherin was found to be decreased, indicating the contribution of these AQP isoforms to EMT, tumor growth and invasion [32]. The correlation of AQP3 and AQP5 with EMT and tumorigenesis has also been reported in triple-negative breast cancer and hepatocellular carcinoma patients, suggesting the potential of AQP3 and AQP5 as prognostic and therapeutic markers [31,75]. Recently, AQP1, AQP3 and AQP5 overexpression was reported to disrupt cell polarity in breast cancer by interacting with the protein Scribble, leading to its downregulation [76,77].

Furthermore, our group reported that both AQP3 and AQP5 play a critical role in cancer cell migration in pancreatic cancer where cells silenced for AQP3 and AQP5 and those double-silenced (AQP3/AQP5) exhibited a significant impairment in cell migration [78,79]. Interestingly, AQP3-, AQP5- and double-silenced human pancreatic ductal adenocarcinoma cells exhibited morphological alterations and reduced cell–cell adhesion, with AQP5 additionally affecting cell stiffness and membrane fluidity. These findings suggest that AQP3 and AQP5 may influence tumor initiation and progression by modulating cellular morphological and biomechanical properties [79].

### 2.2. Peroxiporin Activity of AQP1, AQP3 and AQP5

H_2_O_2_ is the main reactive oxygen species (ROS) acting as a signaling molecule in redox signaling [80]. At low concentrations, H_2_O_2_ can regulate several physiological processes such as cell differentiation, proliferation, survival and immune response via the reversible oxidation of specific protein targets and consequent alteration of their activity [81]. However, at high concentrations, H_2_O_2_ promotes the damage of biomolecules such as proteins, lipids and nucleic acids, and leads to abnormal induction of signaling pathways due to oxidative stress, suggesting its key role in tumorigenesis [82,83,84]. Therefore, the role of AQP1, AQP3 and AQP5 in tumor development is closely associated with their peroxiporin function [12,85]. For instance, AQP3-mediated H_2_O_2_ uptake was correlated with the activation of the EGFR signaling pathway in human squamous cell carcinoma and lung cancer cell lines, contributing to cancer progression [86]. In human breast cancer cell lines, AQP3 peroxiporin activity was found to be implicated in CXCL12/CXCR4-dependent breast cancer cell migration. Extracellular H_2_O_2_ produced by the CXCL12-activated membrane NADPH oxidase 2 (NOX2) is taken up by breast cancer cells through AQP3 with consequent activation of H_2_O_2_-mediated signaling pathways and stimulation of cancer cell migration [86]. Similarly, AQP3 contributes to cervical cancer infiltration and metastasis by mediating NOX4-derived H_2_O_2_ transport and activating the Syk/PI3K/Akt signaling pathway [87]. Moreover, H_2_O_2_ transport via AQP3 can promote tissue inflammation through the NF-ĸB signaling pathway and macrophage activation [88], contributing to the inflammatory response [20,89]. The inhibition of AQP3 peroxiporin activity in melanoma cell lines was recently reported, impairing cancer cell migration, proliferation and adhesion and highlighting the impact of AQP3 on melanoma progression [57].

The involvement of AQP5 in intracellular signaling pathways implicated in tumorigenesis may be attributed to its ability to facilitate H_2_O_2_ transport across cell membranes [67]. Interestingly, AQP5 is often found phosphorylated in cancer as opposed to normal cells, indicating that AQP5 phosphorylation could be related to its role in tumor initiation and development [90]. Moreover, yeast cells transformed to express AQP5 showed increased sensitivity to oxidative damage at long-term exposure to H_2_O_2_, while in the short term, AQP5-mediated H_2_O_2_ permeability was correlated with cell growth and survival. In addition, AQP5 overexpression in pancreatic cancer cells was found to promote cancer cell migration due to its peroxiporin activity [78].

The contribution of AQP1, AQP3 and AQP5 to oxidative stress resistance has been investigated in colon and breast cancer cell lines by assessing their expression before and after treatment with H_2_O_2_. Regarding colorectal cancer, the HT-29 cell line exhibited the highest expression of the three aquaporin isoforms, with no significant changes following H_2_O_2_ exposure, similar to the SW620 cell line. In contrast, H_2_O_2_ treatment upregulated AQP5 in Caco-2 cells and increased AQP1 and AQP3 expression in HCT116 cells [91]. In breast cancer, H_2_O_2_ treatment increased AQP3 expression in MCF7 (estrogen- and progesterone-receptor-positive) and SkBr3 (HER2-positive) cells but downregulated AQP3 in SUM159 (triple-negative) cells, indicating that these cells are less responsive to oxidative stress which aligns with their more aggressive and metastatic phenotype. AQP1 and AQP5 expression decreased in MCF7 cells and was upregulated in SkBr3 and SUM159 cells after the addition of H_2_O_2_ [92]. These findings suggest an adaptive role of aquaporins to stress, which can contribute to the malignancy of tumors and resistance to therapy.

## 3. Role of AQP1, AQP3 and AQP5 as Transceptors in Cancer

In addition to their transport activity, the overexpression of AQP1, AQP3 and AQP5 in cancer has been associated with signal transduction pathways and cytoskeleton reorganization, resembling mechanisms typically mediated by receptors [93]. In fact, AQP1 has been reported to interact with focal adhesion kinases, promoting cell migration in chick neural crest cells and bone marrow mesenchymal stem cells [94,95], and to modulate actin cytoskeleton reorganization by interacting with Lin-7/β-catenin in human endothelial and melanoma cells [96]. Additionally, in estrogen receptor (ER)-positive breast cancer cells, upregulation of AQP3 contributes to cell migration and invasion through the modulation of EMT-related markers and reorganization of the actin cytoskeleton [97]. AQP3 co-localizes with ezrin, influencing both ezrin and actin organization, as well as the formation of lamellipodia and filopodia, with further impact on cell migration and invasion in endometrial epithelial cells [98]. Similarly, AQP5 interacts with ezrin, forming complexes that are mislocated in the salivary glands of Sjögren’s syndrome patients [99]. Interestingly, the trafficking of AQP5 to the plasma membrane depends on the interaction between AQP5-containing vesicles and the cytoskeleton [100,101], a mechanism also observed for AQP1 [102]. Furthermore, AQP5 was found to promote microtubule assembly and stabilization, with a potential impact on paracellular permeability in human airway epithelial cells [103].

The transceptor activity of AQP1, AQP3 and AQP5 could also be related to their interactions with other proteins. For instance, AQP1 interacts with the amyloid precursor protein, which can upregulate AQP1 expression in Alzheimer’s disease patients’ brains [104]. AQP3 forms complexes with ClC-3 chloride channels, regulating ClC-3 channel gating, with both proteins involved in cell volume regulation in nasopharyngeal carcinoma cells [105,106]. Moreover, AQP3 binds to phospholipase D2 (PLD2) in keratinocytes, facilitating glycerol transport to support PLD2-mediated phosphatidylglycerol synthesis [107]. In extravillous trophoblast cells, the disruption of the complex AQP3-caveolin-1 can contribute to pregnancy disorders [108]. In addition, AQP3 can interact with the lipid droplet protein perilipin 1 (PLIN1) [109]. Regarding AQP5-interacting partners, AQP5 co-localizes with the Na^+^-K^+^-Cl^−^ co-transporter 1 (NKCC1) and anion exchanger 2 (AE2) in mouse salivary gland acinar cells and human embryonic kidney (HEK293) cells [110]. Similar to ezrin, prolactin-inducible protein (PIP) can interact with AQP5 [111], controlling its localization in Sjögren’s syndrome patients’ salivary glands [112]. In addition, AQP5 interacts with MUC5AC in rabbit and mouse conjunctival epithelial cells, both being upregulated during acute dry eye stress [113]. In HEK293 cells, AQP5 interaction with ZO-1, plakoglobin, β-catenin and desmoglein-2 resulted in their decreased expression [114]. Importantly, phosphorylated AQP5 binds to c-Src in non-small cell lung cancer cells, promoting EMT [90].

**Figure 3:**
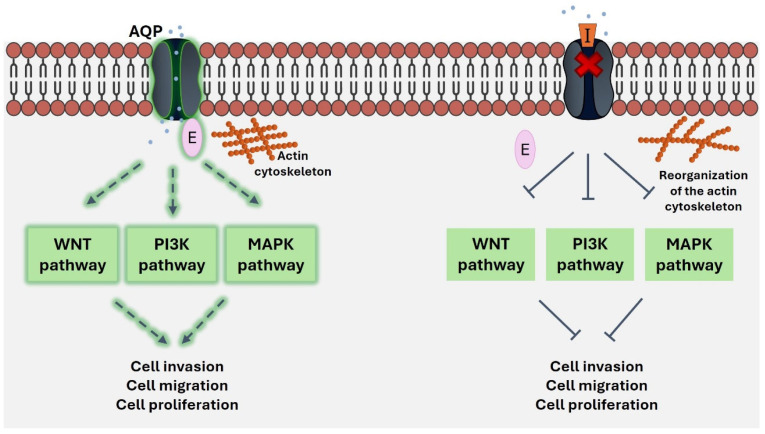
A schematic representation of the role of AQPs as transceptors in cancer. AQPs are overexpressed in cancer cells, contributing to tumor progression through the modulation of key signaling pathways, including Wnt, PI3K and MAPK, and by interacting with the cytoskeleton, probably through the activation of their receptor activity by effector molecules (E). Targeting AQPs with specific inhibitors (I) can disrupt actin cytoskeleton organization and impair the activation of the downstream effectors of cancer signaling, thereby affecting tumor growth and spread. Arrows on the left represent the activation of signaling pathways and tumorigenic processes, while arrows on the right indicate their inhibition. The red “X” represents the blockage of AQP activity.

These findings indicate a potential role of AQPs as transceptors, acting both as transporters and receptors in the cell membranes [93]. This novel concept for aquaporins can be explored by investigating their interplay with signaling pathways and cytoskeleton elements and the identification of novel potent and selective AQP modulators that can impact tumor growth and development (Figure 3).

## 4. AQP1, AQP3 and AQP5 as Druggable Targets for Pharmacological Modulators

Considering their involvement in a wide array of diseases, AQPs have emerged as promising targets for drug discovery, prompting the identification of new potent and selective AQP modulators with therapeutic potential. However, the discovery of selective AQP inhibitors has proven to be very challenging due to the high similarity between AQP isoforms, their widespread expression across different types of tissues and cells and the difficulty of targeting their narrow pores [25,115]. Several pharmacological molecules have been reported to modulate AQP activity and/or expression, demonstrating their therapeutic effects on diseases with abnormal AQP expression (Table 1).

Mercury compounds, such as HgCl_2_, have been found to inhibit AQP1 by binding to the Cys189 residue and preventing the passage of water molecules [116] and were also reported to inhibit other AQPs, including AQP3 and AQP5 [78,117,118]. Interestingly, AQP3 inhibition by HgCl_2_ can improve the sensitivity of prostate cancer cells to cryotherapy [119]. However, the high toxicity and non-specificity of these compounds renders them unsuitable for therapeutic applications [120,121]. In addition, silver compounds, including silver nitrate and silver sulfadiazine, have demonstrated a rapid and irreversible inhibition of AQP1 [122]. Organogold compounds have emerged as potent AQP inhibitors, specifically targeting aquaglyceroporins such as AQP3 and AQP10. In particular, the gold(III) complex [Au(phen)Cl_2_]Cl (phen = 1,10-phenantroline, Auphen) [123] was reported to decrease cell proliferation in AQP3-expressing cells [124] and exhibited an inhibitory effect in red blood cells that endogenously express AQP3, with an IC_50_ of 0.8 ± 0.08 µM [123]. The binding mechanism is likely to occur at the Cys40 residue of AQP3, leading to a conformational change of the protein and consequent inhibition of glycerol permeability [125]. This gold compound was also found to affect AQP7 activity in adipocytes [126,127]. Auphen was reported to exert a therapeutic effect in several pathologies, including hepatocellular carcinoma [128] and inflammation [89], where AQP3 plays an important role. Recently, a new gold compound derivative with the general formula [Au(C^N)Cl_2_] (C^N = cyclometalated ligand) was found to irreversibly inhibit human AQP10 (hAQP10) in a yeast cell model overexpressing this AQP through the formation of a gold adduct followed by cysteine arylation, leading to the inhibition of AQP10-mediated glycerol transport [129]. Moreover, this new class of organogold compounds has demonstrated strong inhibition of AQP3 peroxiporin activity in melanoma cell lines, leading to an impairment in melanoma cell adhesion, cell proliferation and cell migration [57]. Remarkably, these compounds have also exhibited anticancer activity *in vivo* [130]. Recently, the organogold complex ST004 was identified as an AQP3 inhibitor, with its liposomal formulation demonstrating anticancer activity against melanomas in both in vitro and *in vivo* models [131]. Metallodrugs such as [Cu(phen)Cl_2_] (Cuphen) and polyoxotungstate P_2_W_18_ have also been described as potent AQP3 inhibitors, showing anticancer properties [132,133,134,135].

Alongside metallodrugs, small molecules have been validated as potential AQP modulators. Quaternary ammonium ion tetraethylammonium (TEA) and acetazolamide were identified as AQP1 inhibitors in AQP1-expressing *Xenopus laevis* oocytes [136,137] while bumetanide derivatives (AqB013 and AqB011) act as AQP1 blockers [138,139] and furosemide derivative AqF026 functions as an AQP1 activator [140]. However, the modulation of AQP1 activity by TEA, acetazolamide, AqB013 and AqF026 was not validated in other cell models, disclosing the importance of confirming AQP modulation in various AQP expression systems [141,142]. Ciglitazone, a peroxisome proliferator-activated receptor gamma (PPARγ) activator; suberanilohydroxamic acid (SAHA), a histone deacetylase (HDAC) inhibitor; and monomethylfumarate (MMF), an antipsoriatic agent, were reported to increase AQP3 expression levels in human and mouse keratinocytes, promoting glycerol uptake [143,144,145], with SAHA also upregulating AQP5 expression in mouse lung epithelial cells [146]. Bisacodyl decreased AQP3 expression in rat colons, leading to an increase in fecal water content [147]. Moreover, compounds DFP00173 and Z433927330 were shown as potent AQP3 and AQP7 inhibitors, exerting their inhibitory effect through the AQP cytoplasmic entrance [148]. Recently, DFP00173 was found to reduce multiple myeloma cell viability, tumor growth, mitochondrial respiration and electron transport chain complex I activity through AQP3 blockage [149]. Niclosamide was found to upregulate AQP5 expression, which could be beneficial in pathologies with reduced AQP5 levels such as acute lung injury [150], while methazolamide was reported to reduce AQP5 expression, with an impact on the migration of immune cells [151].

Several natural compounds have been identified as modulators of AQP expression. Bacopaside II blocks AQP1-mediated H_2_O_2_ permeability, decreasing the stress-induced hypertrophic remodeling of the heart [152]. All-trans retinoic acid (atRA), chrysin and glycolic acid can reverse the UV-induced reduction in AQP3 expression in human keratinocytes, highlighting their protective role against photoaging [153,154,155], with both atRA and chrysin upregulating AQP3 expression through the ERK and redox signaling pathways [153,154]. In human keratinocytes, resveratrol can reduce AQP3 expression via SIRT1/ARNT/ERK signaling, decreasing cell proliferation [156] while 18β-glycyrrhetinic acid derivative upregulates AQP3 and stimulating dermal fibroblast proliferation and migration [157]. In addition, curcumin inhibits EGF-induced AQP3 upregulation, impairing ovarian cancer cell migration [158]. Daiokanzoto and a resin glycoside fraction from Pharbitis Semen (RFP) reduce AQP3 expression in rat colons, inducing diarrhea [159,160], while naringenin upregulates AQP3 expression levels in a mouse model of constipation [161]. Additionally, β-patchoulene was found to decrease AQP3 expression levels in rats with induced intestinal mucositis through inactivation of the cAMP/PKA/CREB signaling pathway [162]. Recently, we identified rottlerin as an AQP3 inhibitor, acting as a stereochemical lid on the AQP3 pore entrance, blocking AQP3-mediated glycerol transport [163].

AQP1, AQP3 and AQP5 expression and function can also be affected by naturally occurring molecules with potential therapeutic effects, including hormones, microRNAs (miRs) and antibodies. Steroid hormones, including estrogen and progesterone, and arachidonic acid were found to significantly increase AQP1 and AQP5 expression in porcine uteri through the modulation of the PKA and MAPK signaling pathways [164,165,166]. AQP3 is also upregulated by estrogen, being implicated in the stimulation of cell migration and invasion in ER-positive breast cancer cells through the modulation of EMT-related factors and the reorganization of the actin cytoskeleton [97] and in the development of the chicken oviduct and ovarian cancer [167]. In fact, both the AQP3 and AQP5 promoter regions contain a functional estrogen response element that can be activated directly by estrogen [97,168]. In addition, Skowronski and collaborators reported that gonadotropins, prolactin and growth hormone upregulate AQP1 levels in porcine ovarian follicular cells [169]. Testosterone was also reported to upregulate AQP1, AQP3 and AQP5 levels in ovariectomized rats [170,171]. Uroguanylin can trigger lipolysis in human visceral adipocytes via the upregulation of lypolisis-related genes such as AQP3, being usually decreased in obese patients with type 2 diabetes [172]. In addition, leptin administration can ameliorate non-alcoholic fatty liver (NAFLD) disease in leptin-deficient mice decreasing AQP3 expression levels [173]. In acute renal failure, erythropoietin was able to hinder the ischemia-induced downregulation of AQP3, impacting the urinary concentrating ability [174]. Furthermore, both AQP3 and AQP5 were upregulated by dexamethasone and ambroxol in human airway epithelial cells, being able to regulate airway hypersecretion, a typical symptom in several pulmonary diseases [175].

Recently, miRs have been highlighted as a promising tool to modulate AQP expression and function, with therapeutic potential in pathologies characterized by AQP dysregulation. miR-320 can reduce AQP1 expression in breast cancer, decreasing anthracycline chemosensitivity and impairing tumor proliferation, migration and invasion [176,177]. Similarly, miR-1226-3p, miR-19a-3p and miR-19b-3p suppress AQP5 expression in breast cancer, contributing to reduced cell migration [178]. miR-874 targets both AQP1 and AQP3 in several pathologies, mitigating inflammation and myocardial disfunction in sepsis by inhibiting AQP1 [179], and downregulating AQP3 expression in various cancers including gastric cancer [180], pancreatic ductal adenocarcinoma [28] and non-small cell lung cancer (NSCLC) [181], impairing tumor development. This miRNA also contributes to intestinal barrier dysfunction via AQP3 modulation [182,183]. miR-185 regulates AQP3 and AQP5 expression, exerting its therapeutic effect by compromising squamous cell carcinoma and colorectal cancer progression [184,185], whereas miR-877 can suppress AQP3 in gastric cancer, promoting apoptosis and reducing cell proliferation, invasion and EMT [186]. In hepatocellular carcinoma, miR-124 can decrease AQP3 expression, inhibiting cell proliferation and migration [60], while miR-1271-5p can prevent hepatitis B-virus-mediated liver cancer growth *in vivo* by decreasing the expression of AQP5 [187]. In addition, miR-21 can downregulate AQP5 in gallbladder carcinoma [188] and miR-29a can increase the intestinal membrane permeability of colonic epithelial cells by reducing AQP1 and AQP3 expression in diarrhea-predominant irritable bowel syndrome (IBS-D) [189]. In disseminated intravascular coagulation, miR-96 and miR-330 can lower AQP5 expression, leading to pulmonary edema [190]. In sepsis, miR-133a-3p downregulates AQP1, resulting in increased expression of inflammatory cytokines [191]. In acute lung injury, miR-126-5p and miR-144-3p were found to reduce AQP1 expression, improving fluid clearance and promoting apoptosis [161,192]. miR-495 can inhibit AQP1 expression through the p38 MAPK signaling pathway, enhancing osteoblast proliferation and differentiation in mice with tibial fractures [193].

The urgent need for specific AQP modulators has prompted efforts to develop antibodies capable of selectively targeting AQP isoforms. An anti-AQP3 monoclonal antibody was designed to target a specific extracellular epitope of AQP3 in macrophages. In a mouse model of liver injury, the anti-AQP3 antibody suppressed inflammation by inhibiting AQP3-mediated H_2_O_2_ permeability, thereby downregulating the NF-ĸB signaling pathway and reducing macrophage activation [88]. Recently, this antibody exhibited therapeutic effects in colorectal cancer by suppressing tumor growth, reducing immunosuppressive M2-like tumor-associated macrophages, and maintaining T cell anti-tumor activity within the tumor microenvironment through AQP3 targeting [194]. In addition, the anti-AQP3 monoclonal antibody was found to reduce multiple myeloma cell viability, tumor growth, mitochondrial respiration and electron transport chain complex I activity via AQP3 inhibition [149]. These findings disclose the pivotal role of AQP3 in oxidative stress, inflammation and cancer, while also highlighting the potential of antibody-based therapies for the treatment of AQP-overexpressing pathologies.

Further research is needed to address the current challenges in AQP druggability by improving the selectivity and efficiency of aquaporin modulators and developing targeted therapies for their precise delivery to cancer tissues. Significant progress has been made via the development of anti-AQP antibodies [88] and via the encapsulation of AQP3 inhibitors such as Cuphen [132,134] and ST004 [131] in liposomal formulations designed to target cancer tissues. In addition, the development of AQP modulators that selectively affect AQP receptor activity without influencing their transporter function would significantly improve our understanding of their roles in cancer development and metastasis.

**Table 1:** Modulators of AQP1 AQP3 and AQP5 expression and function.

AQP	Modulator	Effect on AQP	Targeted Disease	References
AQP1	HgCl_2_	Inhibition	-	[116]
	Silver nitrate	Inhibition	-	[122]
	Silver sulfadiazine	Inhibition	-	[122]
	AqB011	Inhibition	Colon cancer	[139]
	Bacopaside II	Inhibition	Cardiac hypertrophy	[152]
	Estrogen	Upregulation	-	[164,165,166]
	Progesterone	Upregulation	-	[164,165,166]
	Arachidonic acid	Upregulation	-	[164,165,166]
	Gonadotropins	Upregulation	-	[169]
	Prolactin	Upregulation	-	[169]
	Growth hormone	Upregulation	-	[169]
	Testosterone	Upregulation	-	[171]
	miR-29a	Downregulation	IBS-D	[189]
	miR-126-5p	Downregulation	Acute lung injury	[192]
	miR-133a-3p	Downregulation	Sepsis	[191]
	miR-144-3p	Downregulation	Acute lung injury	[195]
	miR-320	Downregulation	Breast cancer	[176,177]
	miR-495	Downregulation	-	[193]
	miR-874	Downregulation	Sepsis	[179]
AQP3	HgCl_2_	Inhibition	Prostate cancer	[117,118,119]
	Auphen	Inhibition	Epidermoid carcinoma; hepatocellular carcinoma; inflammation; melanoma; triple-negative breast cancer	[57,89,123,124,125,128,130]
	Au(III) C^NH^N	Inhibition	Melanoma	[57]
	Au(III) C^CO^N	Inhibition	Melanoma	[57]
	ST004	Inhibition	Melanoma	[131]
	Cuphen	Inhibition	Melanoma; colon cancer	[132,133,134]
	P_2_W_18_	Inhibition	Melanoma	[135]
	Ciglitazone	Upregulation	-	[143]
	SAHA	Upregulation	-	[144]
	MMF	Upregulation	Psoriasis	[145]
	Bisacodyl	Downregulation	Constipation	[147]
	DFP00173	Inhibition	Multiple myeloma	[148,149]
	atRA	Upregulation	Skin photoaging	[153]
	Chrysin	Upregulation	Skin photoaging	[154]
	Glycolic acid	Upregulation	Skin photoaging	[155]
	Resveratrol	Downregulation	-	[156]
	18β-glycyrrhetinic acid	Upregulation	-	[157]
	Curcumin	Downregulation	Ovarian cancer	[158]
	Daiokanzoto	Downregulation	Constipation	[159]
	RFP	Downregulation	Constipation	[160]
	Naringenin	Upregulation	Constipation	[161]
	β-patchoulene	Downregulation	Intestinal mucositis	[162]
	Rottlerin	Inhibition	-	[163]
	Estrogen	Upregulation	ER-positive breast cancer; ovarian cancer	[97,167]
	Testosterone	Upregulation	-	[170]
	Uroguanylin	Upregulation	-	[172]
	Leptin	Downregulation	Obesity; NAFLD	[173]
	Erythropoietin	Upregulation	Acute renal failure	[174]
	Dexamethasone	Upregulation	-	[175]
	Ambroxol	Upregulation	-	[175]
	miR-29a	Downregulation	IBS-D	[189]
	miR-124	Downregulation	Hepatocellular carcinoma	[60]
	miR-185	Downregulation	Squamous cell carcinoma	[184]
	miR-874	Downregulation	Gastric cancer; pancreatic ductal adenocarcinoma; NSCLC; intestinal barrier dysfunction	[28,180,181,182,183]
	miR-877	Downregulation	Gastric cancer	[186]
	Anti-AQP3 monoclonal antibody	Inhibition	Liver injury; colorectal cancer; multiple myeloma	[88,149,194]
AQP5	HgCl_2_	Inhibition	Pancreatic ductal adenocarcinoma	[78]
	SAHA	Upregulation	-	[146]
	Niclosamide	Upregulation	-	[150]
	Methazolamide	Downregulation	Sepsis	[151]
	Estrogen	Upregulation	-	[164,165,166]
	Progesterone	Upregulation	-	[164,165,166]
	Arachidonic acid	Upregulation	-	[164,165,166]
	Testosterone	Upregulation	-	[171]
	Dexamethasone	Upregulation	-	[175]
	Ambroxol	Upregulation	-	[175]
	miR-19a-3p	Downregulation	Breast cancer	[178]
	miR-19b-3p	Downregulation	Breast cancer	[178]
	miR-21	Downregulation	Gallbladder carcinoma	[188]
	miR-96	Downregulation	Disseminated intravascular coagulation	[190]
	miR-185	Downregulation	Colorectal cancer	[185]
	miR-330	Downregulation	Disseminated intravascular coagulation	[190]
	miR-1226-3p	Downregulation	Breast cancer	[178]
	miR-1271-5p	Downregulation	Hepatitis B-virus-mediated liver cancer	[187]

## 5. Final Remarks

This review focuses on the pivotal roles of aquaporins AQP1, AQP3 and AQP5 in tumor biology, emphasizing their significant contributions to cancer progression. The overexpression of these aquaporins has been strongly associated with enhanced tumor growth, increased metastatic potential and poor clinical outcomes in patients. While traditionally recognized for their primary role in facilitating the transport of water, glycerol and other small molecules, such as H_2_O_2_, across cellular membranes, recent studies have unveiled their broader functionality. These proteins also act as transceptors, integrating transport and signaling roles to mediate signal transduction and the activation of critical pathways involved in tumor initiation and progression.

Given their multifaceted contributions to cancer biology, tumor aquaporins emerge as compelling therapeutic targets. Their involvement extends beyond basic transport functions to include regulatory roles in key oncogenic signaling cascades. This dual functionality underscores the need for innovative therapeutic strategies aimed at modulating their activity. Consequently, there is a growing interest in designing and identifying novel, potent and selective modulators capable of targeting the unique properties of AQP-overexpressing tumors. By addressing the challenges associated with their dysregulation, these advancements could pave the way for more effective treatments and improved prognoses for patients with AQP-driven cancers.

## Data Availability

No new data were created or analyzed in this study.
